# Development of a sweetened beverage tax, Philippines

**DOI:** 10.2471/BLT.18.220459

**Published:** 2018-12-01

**Authors:** Frances Claire C Onagan, Beverly Lorraine C Ho, Karl Kendrick T Chua

**Affiliations:** a Health Policy Development and Planning Bureau, Building 3, Department of Health, San Lazaro Compound, Rizal Avenue, Santa Cruz, Manila, 1003, Philippines.; b Health Policy Development and Planning Bureau, Department of Health, Manila, Philippines.; c Strategy, Economics and Results Group, Department of Finance, Manila, Philippines.

## Abstract

**Problem:**

Both sugar-sweetened beverage consumption and the incidence of obesity have increased in the Philippines in recent years.

**Approach:**

A proposal to tax sugar-sweetened beverages was introduced in the House of Representatives and merged into a proposed comprehensive Tax Reform for Acceleration and Inclusion (TRAIN) Bill to increase the likelihood of acceptance. The health department and finance department recommended a policy that would maximize benefits to both public health and government revenue. To advance discussions, the health department expanded the health argument to include the country’s poor performance in oral health. The approved TRAIN Law adopted the term sweetened beverage to emphasize that the tax covers both sugar and non-sugar sweetened beverages. The tax rate was set to 6.00 Philippine pesos (0.111 United States dollars) per litre of sweetened beverages. The sugar industry successfully lobbied for higher tax rates on beverages containing high-fructose corn syrup, resulting in a differential rate of 12.00 Philippine pesos per litre.

**Local setting:**

Despite a 12% value-added tax on sugar-sweetened beverages, sales had been sustained by enhanced marketing and product variants being offered in small portions.

**Relevant changes:**

One month after implementation of the tax in 1 January 2018, prices of taxable sweetened beverages had increased by 16.6 to 20.6% and sales in *sari-sari* (convenience) stores had declined 8.7%.

**Lessons learnt:**

The tax benefited from high-level government commitment and support, keeping policy simple reduced opportunities for tax avoidance and evasion, and taking both health and non-health considerations into account were helpful in arguing for the tax.

## Résumé

**Problème:**

Au cours des dernières années, les Philippines ont enregistré une augmentation de la consommation de boissons sucrées avec du sucre et de l'incidence de l'obésité.

**Approche:**

Une proposition de taxe sur les boissons sucrées avec du sucre a été présentée à la Chambre des représentants et a engendré un projet de loi détaillé appelé « Tax Reform for Acceleration and Inclusion » (TRAIN) destiné à accroître la probabilité d'acceptation. Le ministère de la Santé et le ministère des Finances ont recommandé l'adoption d'une politique qui présenterait des avantages en matière de santé publique, mais aussi pour les recettes de l'État. Afin de faire avancer les discussions, le ministère de la Santé a élargi le débat sur la santé de façon à inclure les mauvais résultats du pays en matière de santé bucco-dentaire. La loi TRAIN utilise l'expression « boisson sucrée » pour souligner le fait que la taxe s'applique à la fois aux boissons sucrées avec du sucre et aux boissons sucrées avec d'autres substances. Le taux de la taxe a été fixé à 6,00 pesos philippins (0,111 dollar des États-Unis) par litre de boisson sucrée. L'industrie du sucre a plaidé avec succès pour l'application d'un taux plus important aux boissons contenant du sirop de maïs à haute teneur en fructose, ce qui a donné lieu à un taux différencié de 12,00 pesos philippins par litre.

**Environnement local:**

Malgré une taxe sur la valeur ajoutée de 12% sur les boissons sucrées avec du sucre, les ventes se sont maintenues grâce à un marketing renforcé et à des variantes de produits proposés en petites portions.

**Changements significatifs:**

Un mois après l'instauration de la taxe le 1er janvier 2018, les prix des boissons sucrées imposables avaient augmenté de 16,6 à 20,6% et les ventes dans les magasins *sari-sari* (de proximité) avaient diminué de 8,7%.

**Leçons tirées:**

La taxe a bénéficié d'un engagement et d'un soutien de haut niveau de la part du gouvernement; le fait que la politique soit simple a réduit les possibilités d'évasion et de fraude fiscales et la prise en compte de considérations aussi bien sanitaires que non sanitaires a été utile dans l'argumentaire en faveur de la taxe.

## Resumen

**Problema:**

Tanto el consumo de bebidas azucaradas como la incidencia de la obesidad han aumentado en Filipinas en los últimos años.

**Enfoque:**

Se presentó una propuesta de aplicar un impuesto a las bebidas azucaradas en la Cámara de Representantes y se fusionó en una propuesta de Reforma Tributaria integral para el Proyecto de Ley de Aceleración e Inclusión (TRAIN, por sus siglas en inglés) para aumentar la probabilidad de aceptación. El departamento de salud y el departamento financiero recomendaron una política que maximice los beneficios tanto para la salud pública como para los ingresos del gobierno. Para avanzar en las discusiones, el departamento de salud amplió el argumento de la salud para incluir el pobre desempeño del país en salud bucal. La Ley TRAIN aprobada adoptó el término «bebidas endulzadas» para enfatizar que el impuesto cubre tanto las bebidas endulzadas con y sin azúcar. El impuesto se fijó en 6,00 pesos filipinos (0,111 dólares estadounidenses) por litro de bebidas endulzadas. La industria azucarera presionó con éxito para que se aplicaran tasas impositivas más altas a las bebidas que contienen jarabe de maíz con un alto contenido de fructosa, lo que resultó en una tasa diferencial de 12,00 pesos filipinos por litro.

**Marco regional:**

A pesar de un impuesto al valor añadido del 12 % sobre las bebidas azucaradas, las ventas se habían mantenido gracias a la mejora de la comercialización y a las variantes de productos que se ofrecían en pequeñas porciones.

**Cambios importantes:**

Un mes después de la implementación del impuesto, el 1 de enero de 2018, los precios de las bebidas endulzadas imponibles habían aumentado entre un 16,6 y un 20,6 % y las ventas en las tiendas *sari-sari* (supermercados) habían disminuido un 8,7 %.

**Lecciones aprendidas:**

El impuesto se benefició del compromiso y el apoyo de alto nivel del gobierno, que mantuvo la política simple, lo que redujo las oportunidades de evasión y elusión de impuestos, y tuvo en cuenta tanto las consideraciones de salud como las no relacionadas con la misma, lo que fue de gran ayuda para argumentar a favor del impuesto.

## ملخص

المشكلة ازداد كل من استهلاك المشروبات المحلاة بالسكر ومعدل السمنة في الفلبين في السنوات الأخيرة.

الأسلوب تم تقديم اقتراح لفرض ضريبة على المشروبات المحلاة بالسكر في مجلس النواب ودمجها في مشروع قانون الإصلاح الضريبي الشامل للتعجيل والإدماج (TRAIN)، وذلك بهدف زيادة احتمالات القبول. وأوصت كل من وزارة الصحة ووزارة المالية بسياسة من شأنها تعظيم الفوائد بالنسبة للصحة العامة والإيرادات الحكومية. ولدفع التقدم في المناقشات، قامت وزارة الصحة بتوسيع حجم النقاش الصحي ليشمل الأداء المترهل للدولة في مجال صحة الفم. اعتمد قانون TRAIN الذي نال الموافقة على مصطلح المشروبات المحلاة للتأكيد على أن الضريبة تغطي كلا من المشروبات المحلاة أو غير المحلاة بالسكر. تم ضبط معدل الضريبة على 6.00 بيزو فلبيني (0.111 دولار أمريكي) لكل لتر من المشروبات المحلاة. مارس قطاع صناعة السكر ضغوطاً ناجحة من أجل رفع معدلات الضريبة على المشروبات التي تحتوي على شراب الذرة بنسبة عالية من الفركتوز، مما أدى إلى معدل تفاضلي قدره 12.00 بيزو فلبيني لكل لتر.

المواقع المحلية على الرغم من فرض ضريبة للقيمة المضافة بنسبة 12٪ على المشروبات المحلاة بالسكر، إلا أن المبيعات قد ازدادت من خلال التسويق المحسّن ومتغيرات المنتج الذي يتم تقديمه في أحجام صغيرة.

التغيّرات ذات الصلة بعد شهر واحد من تنفيذ الضريبة في 1 يناير/كانون ثاني 2018، كانت أسعار المشروبات المحلاة الخاضعة للضريبة قد ارتفعت بنسبة 16.6 إلى 20.6٪، وانخفضت المبيعات في متاجر ساري-ساري (محلات البقالة الصغيرة) بنسبة 8.7٪.

الدروس المستفادة استفادت الضريبة من الالتزام والدعم رفيع المستوى من الحكومة، كما أن السياسة البسيطة المستخدمة أدت إلى الحد من فرص التهرب من الضريبة وتجنبها، كذلك فإن وضع الاعتبارات الصحية وغير الصحية في الحسبان كان مفيداً في الترويج للضريبة.

## 摘要

**问题:**

近年来，菲律宾含糖饮料的销量和肥胖率均有增长。

**方法:**

众议院提出了对含糖饮料征税的提案，并将之纳入提议的全面的《税制改革加速包容法案 (TRAIN)》中，以提高其被接受的可能性。卫生部门和财政部门提出一项政策，旨在最大限度地提高公共卫生和政府收入的效益。为了推进讨论，卫生部门拓展了健康论点，将该国口腔卫生状况不佳的情况纳入讨论。获批的 TRAIN 采用“含糖饮料”这一术语，强调了此项税收包括含糖饮料和无糖甜味饮料。含糖饮料的税率为每升 6.00 菲律宾比索（0.111 美元）。对于含有高果糖玉米糖浆的饮料，制糖业成功游说对其征收更高的含糖税，即税率达到每升 12.00 菲律宾比索。

**当地状况:**

尽管对含糖饮料征收了 12% 的增值税，但得益于市场营销和少量的产品变型，其销量仍得以维持。

**相关变化:**

自 2018 年 1 月 1 日起实施该税法一个月后，需缴税的含糖饮料价格上升了 16.6% 至 20.6% 而 *sari-sari* （便利店）的销量降低了 8.7%。

**经验教训:**

该税法得益于政府高层的承诺和支持，简单明晰的政策规定降低了避税、逃税的机会，并将有利于税收讨论的健康和非健康因素均纳入考量。****

## Резюме

**Проблема:**

В последние годы на Филиппинах увеличилось потребление сахаросодержащих напитков, а также количество людей, страдающих ожирением.

**Подход:**

В Палату представителей было внесено предложение о введении налога на сахаросодержащие напитки, которое было добавлено в законопроект о налоговой реформе (Tax Reform for Acceleration and Inclusion, TRAIN), чтобы увеличить вероятность принятия этого закона. Департаменты здравоохранения и финансов рекомендовали подход, который бы максимально увеличивал пользу как для общественного здравоохранения, так и для государственных доходов. В ходе дальнейших обсуждений департамент здравоохранения расширил свои аргументы, включив в них плохие показатели состояния здоровья полости рта в среднем по стране. В принятом законе TRAIN используется термин «сладкий напиток», чтобы подчеркнуть, что налог распространяется на все сладкие напитки как с содержанием сахара, так и с его заменителями. Налоговая ставка была установлена в размере 6,00 филиппинского песо (0,111 доллара США) на литр сладкого напитка. Сахарная промышленность успешно лоббировала применение более высоких налоговых ставок на напитки, содержащие кукурузный сироп с высоким содержанием фруктозы, что привело к разнице в 12,00 филиппинского песо на литр напитка.

**Местные условия:**

Несмотря на 12%-й налог на добавленную стоимость на сахаросодержащие напитки, их продажи поддерживались за счет активного маркетинга и возможности приобретения небольших порций продукции.

**Осуществленные перемены:**

Первого января 2018 года, через месяц после введения налога, цены на облагаемые налогом сладкие напитки возросли на 16,6–20,6%, а продажи в магазинах *sari-sari* (небольшие бакалейные магазины или киоски) упали на 8,7%.

**Выводы:**

Введение налога принесло пользу благодаря поддержке правительства и соблюдению им своих обязательств, простоте подхода и сокращению возможностей для уклонения от уплаты налогов. Обсуждению налога помогло то, что при этом принимались во внимание как факторы в области здравоохранения, так и факторы, не связанные с этой областью.

## Introduction

Sugar-sweetened beverages are more strongly associated with high energy intake and weight gain than any other form of processed food.^1^ In the Philippines, both the proportion of the population that consumes these beverages and per capita consumption increase with age (Pulse Asia Research Inc., unpublished report, 2017). Moreover, the fraction of daily sugar intake that comes from sugar-sweetened beverages increased 44% in 10 years: in 2005, Filipinos consumed 14.9 g of sugar per capita per day from sugar-sweetened beverages alone; in 2015, it was 21.4 g (M Abrigo and K Francisco, Philippine Institute for Development Studies, unpublished report, 2018).

Obesity prevalence in the Philippines have remained low relative to other countries in the Association of South-East Asian Nations (ASEAN).^2^ Nevertheless, a growing proportion of Filipinos of all ages are overweight or obese, which is likely to substantially increase the number of productive years lost due to poor health.^3^ Cross-country comparisons among ASEAN member states indicate that the loss of productive years due to obesity is greatest in the Philippines.^4^ The annual cost of obesity-related productivity loss in the country has been estimated to 567 million United States dollars.^2^

## Local setting

Before 1 January 2018, no specific tax applied to sugar-sweetened beverages in the Philippines, although they were subject to a general 12% value-added tax. Beverage manufacturers sustained sales by enhanced marketing and offering products in small portion, this lowered the unit price of sugar-sweetened beverages (Organic Intelligence Consulting Inc., unpublished report, 2017) and increased the likelihood of frequent consumption.^5^ When the World Health Organization recommended taxes on sugar-sweetened beverages to address childhood obesity in 2016,^6^ the Philippines was presented with the opportunity to enact another landmark piece of health legislation to follow the 2012 Sin Tax Reform Law on Tobacco and Alcohol.^7^ A proposal to tax sugar-sweetened beverages was filed by a first-term lawmaker in the House of Representatives (House Bill 3365) during the 16^th^ Congress (from 2013 to 2016).^8^ When this was not successful, she refilled it (House Bill 292) during the 17^th^ Congress (from 2016 to 2019) and then secured the support of the health department and the finance department. This partnership between executive and legislative branches of the government culminated in the Philippines, becoming the third ASEAN member state after Brunei Darussalam and Thailand to impose taxes on sugar-sweetened beverages.

## Legislative approach

As the sugar-sweetened beverage tax was a health-related tax, the health department and finance department collaborated on recommending a tax policy that would maximize benefits to both public health and government revenue and that considered reviews of the best available evidence, including in-house evidence from both agencies. In particular, the policy focused on firstly modifying health risks by introducing taxes that increased the price of sugar-sweetened beverages sufficiently to deter purchases and that could be applied to a wide range of products, thereby discouraging unhealthy substitution. Secondly the policy also focused on securing revenues by using a unitary tax scheme (i.e. applying a single tax rate) and volumetric tax collection (i.e. basing tax on the volume of sugar per litre of beverage), both of which simplify tax administration and minimize opportunities for avoidance and evasion. Box 1 summarizes the legislation’s development.

Box 1 **Legislative steps towards a sweetened beverage tax, the Philippines, 2017**

**House Bill 292 (standalone sugar-sweetened beverage tax bill), included in the TRAIN Bill 26 April 2017**

Definition: sugar-sweetened beverages were defined as non-alcoholic beverages that contain caloric sweeteners or added sugar or artificial or non-caloric sweeteners in the form of a liquid, syrup, concentrate or solid mixture that is added to water or other liquids to make a drink.

Taxable products: (i) soft drinks and carbonated drinks; (ii) fruit drinks and punches; (iii) sports drinks; (iv) sweetened tea and coffee drinks; (v) energy drinks; and (vi) all non-alcoholic beverages that are ready to drink or in powder form and contain added natural or artificial sugar.

Exemptions: (i) 100% natural fruit juices without added sugar or caloric sweeteners; (ii) 100% natural vegetable juices without added sugar or caloric sweeteners; (iii) yogurt and fruit-flavoured yogurt beverages; (iv) meal-replacement beverages (e.g. medical food), weight-loss products, any liquid or powder drink or product for oral nutritional therapy; and (v) all milk products.

Tax rate: 10.00 Philippine pesos^b^ per litre with a 4% increment each year to adjust for inflation.

**House Bill 5636 (House version of the TRAIN Bill) passed 31 May 2017**

Definition: sugar-sweetened beverages were defined as non-alcoholic beverages of any constitution (i.e. liquid, powder or concentrate) that are prepackaged and sealed in accordance with Philippine Food and Drug Administration standards and that contain sugar added by the manufacturers.

Taxable products: (i) sweetened juice drinks; (ii) sweetened tea and coffee; and (iii) other beverages (including all carbonated beverages) with added sugar or caloric or non-caloric sweeteners, flavoured water, energy drinks, sports drinks, powdered drinks not classified as milk, juice, tea or coffee, cereal and grain beverages, and other non-alcoholic beverages that contain added sugar.

Exemptions: (i) plain milk and milk drink products without added sugar; (ii) all milk drink products, infant formula and milk alternatives (e.g. soy milk and almond milk), including flavoured milk, such as chocolate milk; (iii) 100% natural fruit juices without added sugar or caloric sweeteners; (iv) 100% natural vegetable juices without added sugar or caloric sweeteners; (v) meal-replacement and medically indicated beverages; (vi) ground coffee; and (vii) unsweetened tea.

Tax rate: 10.00 Philippine pesos^b^ per litre for beverages containing locally sourced sweeteners and 20.00 Philippine pesos^b^ per litre for beverages containing imported sweeteners.

**Senate Bill 1592 (Senate version of the TRAIN Bill) passed 27 November 2017**

Definition: sweetened beverages were defined as non-alcoholic beverages of any constitution (i.e. liquid, powder or concentrate) that are pre-packaged and sealed in accordance with Philippine Food and Drug Administration standards and that contain caloric or non-caloric sweeteners or both added by the manufacturers.

Taxable products: (i) sweetened juice drinks; (ii) sweetened tea; (iii) all carbonated beverages; (iv) flavoured water; (v) energy and sports drinks; (vi) powdered drinks not classified as milk, juice, tea or coffee; (vii) cereal and grain beverages; and (viii) other non-alcoholic beverages that contain added sugar.

Exemptions: (i) plain milk, infant formula milk and growing-up milk; (ii) powdered milk, ready-to-drink milk, flavoured milk and fermented milk; (iii) 100% natural fruit juices without added sugar or caloric sweeteners; (iv) 100%natural vegetable juices without added sugar or caloric sweeteners; (v) meal-replacement and medically indicated beverages; (vi) ground coffee, instant soluble coffee and prepackaged powdered coffee products, with or without added sugar; (vii) unsweetened tea; and (viii) beverages sweetened with coconut sap or stevia glycosides.

Tax rate: 4.50 Philippine pesos^b^ per litre for beverages sweetened with a caloric or non-caloric sweetener (except high-fructose corn syrup) and 9.00 Philippine pesos^b^ per litre for beverages sweetened with high-fructose corn syrup.

**Republic Act 10963 Section 47 (TRAIN Law) signed into law 19 December 2017**

Definition: sweetened beverages were defined as non-alcoholic beverages of any constitution (i.e. liquid, powder or concentrate) that are prepackaged and sealed in accordance with Philippine Food and Drug Administration standards and that contain caloric or non-caloric sweeteners or both added by the manufacturers.

Taxable products: (i) sweetened juice drinks; (ii) sweetened tea; (iii) all carbonated beverages; (iv) flavoured water; (v) energy and sports drinks; (vi) powdered drinks not classified as milk, juice, tea or coffee; (v) cereal and grain beverages; and (vi) other non-alcoholic beverages that contain added sugar.

Exemptions: (i) all milk products, including plain milk, infant formula milk, follow-on milk, growing-up milk, powdered milk, ready-to-drink milk, flavoured milk, fermented milk, soy milk and flavoured soy milk; (ii) 100% natural fruit juices without added sugar or caloric sweeteners; (iii) 100% natural vegetable juices without added sugar or caloric sweeteners; (iv) meal-replacement and medically indicated beverages; and (v) ground coffee, instant soluble coffee and prepackaged powdered coffee products; and (vi) beverages sweetened with coconut sap or stevia glycosides.

Tax rate: 6.00 Philippine pesos^a^ per litre for beverages sweetened with caloric or non-caloric sweeteners (except high-fructose corn syrup) and 12.00 Philippine pesos^a^ per litre for beverages sweetened with high-fructose corn syrup.

TRAIN: Tax Reform for Acceleration and Inclusion.^a^ In 2017, 1 Philippine peso was equivalent to 0.0185 United States dollars.Note: In the Philippines, fiscal policies requiring legislation follow a sequential process in Congress: (i) tax proposals should pass the House of Representatives before the Senate initiates discussions on their counterpart bill; (ii) differences between versions of the bill passed by the House of Representatives and the Senate are reconciled during a Bicameral Conference; and (iii) both chambers of Congress then ratify the reconciled version, which is sent to the Office of the President to be signed.

The sugar-sweetened beverage tax was framed as a preventive health measure that addressed features of the food market associated with increased rates of obesity and diabetes. The acceptance of the tax was hampered by: (i) limited interest in tackling obesity and diabetes; (ii) the claim that sugar-sweetened beverages help poor people satisfy their dietary needs;^9^ (iii) the misconception that the positive health effects of the tax would favour richer households with more flexible spending power; and (iv) the strongly held belief that undernutrition is the real problem despite evidence of the country’s double burden of malnutrition (i.e. the coexistence of undernutrition and diet-related noncommunicable diseases).^3^ To increase the likelihood that the tax proposal would be passed, it was incorporated into the government’s proposed comprehensive Tax Reform for Acceleration and Inclusion (TRAIN) Bill. Certification of the TRAIN Bill as urgent by the Philippine president was instrumental in ensuring the sugar-sweetened beverage tax entered into law.^10^

To advance discussions, the health argument was expanded to include the country’s poor performance in oral health. Although prolonged sugar exposure has been strongly association with dental caries, this association has not often been used to support sugar-sweetened beverage tax policies. Dental caries are common in the Philippines, with a national prevalence of 88%.^11^ Moreover, untreated dental caries among Filipino children have been linked to being underweight,^12^ and data from the education department indicate that toothache is a principal cause of school absenteeism. This argument contributed to a compelling narrative that helped anchor the sugar-sweetened beverage tax policy within the TRAIN Bill; namely the tax proposal supported human capital development and ongoing universal health-care reforms.

After advancing through both chambers of Congress, the Bicameral Conference Committee reconciled differences between the sweetened beverages tax proposals incorporated in House Bill 5636 and Senate Bill 1592. The TRAIN Law signed by the president adopted most provisions in the Senate version (Box 1), including use of the term “sweetened beverages” to emphasize that the tax covers both sugar and non-sugar sweetened beverages. Successful lobbying by the sugar industry resulted in the decisions: (i) to impose a high differential tax rate on drinks containing high-fructose corn syrup; and (ii) to subject artificially sweetened beverages to an excise tax. The local sugar industry, which had been disadvantaged by an influx of high-fructose corn syrup into the country, expressed concern that food manufacturers would shift to artificial sweeteners should artificially sweetened beverages be exempted from excise tax. The tax on artificially sweetened beverages was also supported by medical societies as a way of reducing consumption of all types of sweetened beverage.

The final tax rate was set to 6.00 Philippine pesos (equivalent to 0.111 United States dollars in 2017) per litre for beverages sweetened with caloric or non-caloric sweeteners, except for beverages sweetened with high-fructose corn syrup with a tax of 12.00 Philippine pesos per litre (Box 1).

## Impact of the tax

A month after the sweetened beverage tax was implemented on 1 January 2018, market surveillance indicated that the average price of taxable sweetened beverages in *sari-sari* stores (i.e. neighbourhood convenience stores) had increased by 20.6% and average prices in supermarkets had increased by 16.6%.^13^ Among taxable product categories, carbonated non-alcoholic drinks experienced the highest average price hike, at 21.0%. *Sari-sari* stores experienced the greatest decline in sales, which averaged 8.7% over the month.^14^ Given that the tax has just recently been implemented, it is too soon to evaluate its impact on risks to population health. A monitoring programme is planned to investigate changes in consumers’ purchasing and consumption behaviour and the food industry’s response. The health department has allocated research funds to start monitoring in 2019.

Implementation of the sweetened beverage tax also catalysed substantial policy changes in the food system. The resulting Implementing Rules and Guidelines meant that prepacked concentrates sold to food retailers for dispensing were also subject to excise tax. As a result, the unlimited beverage refills offered in some food outlets have been discontinued. In addition, the president issued a directive to put health warning labels on sweetened beverages to help consumers make an informed choice.^15^ This provided an opportunity to finally regulate front-of-pack labels and to counter misleading brand messages from manufacturers.

## Lessons learnt

The main lessons learnt in establishing the sweetened beverage tax are summarized in Box 2. First, the tax greatly benefited from visible, high-level, sustained commitment from both legislative and executive branches of government, which counterbalanced opposition led by the beverage industries. In addition, the soft power represented by the presence of former health ministers, incumbent cabinet officials, development partners and legislators at public hearings enhanced the political desirability of the reform. Experience in the Philippines demonstrates that taxes relevant to health do not have to be framed or designed as exclusively health or revenue measures. Moreover, reduced consumption and higher revenues can be sustained over the long term by ensuring taxes are simple to implement administratively. Policies should also be kept simple to avoid loopholes that could provide opportunities for tax avoidance and evasion or for biased interpretations of the legislation that could weaken the tax base. Although obesity is the dominant health rationale globally for imposing sugar-sweetened beverage taxes, the lack of political interest in addressing obesity in the Philippines meant that progress depended on framing the threat to health differently. Taking both health and non-health considerations into account could therefore be valuable when developing comprehensive, highly nuanced and compelling arguments about the societal cost of poor health over the long term.

Box 2 **Summary of main lessons learnt**

- Visible, high-level, government commitment and support were vital for establishing the sweetened beverage tax.
- A simple and clear policy reduced opportunities for tax avoidance and evasion and helped avoid biased interpretations of the legislation that could weaken the tax base.
- Both health and non-health considerations were helpful to take into account in developing comprehensive and compelling arguments for the tax.

This paper describes strategies used in the Philippines that could help other countries develop fiscal interventions to address market failures influencing health. These interventions should balance health and fiscal objectives. Four other ASEAN member states are already planning to implement sugar-sweetened beverage taxes: (i) Indonesia and Singapore are exploring appropriate policy designs; and (ii) Malaysia and Viet Nam are finalizing proposals for submission to lawmakers. Although a sugar-sweetened beverage tax will not reverse the burden of malnutrition and noncommunicable diseases by itself, it could trigger a domino effect in the food system that will modify health risk factors. Such a tax could be a tangible first step towards re-engineering an obesogenic environment by denormalizing the consumption of sugar-sweetened beverages in the mind of the public.
